# Evaluation of eradication efficacy and optimization strategies for *Helicobacter pylori* infection based on real-world data: a multivariable retrospective study

**DOI:** 10.3389/fmed.2026.1868250

**Published:** 2026-07-01

**Authors:** Ziqi He, Linde He, Yaliu Qu, Yiting Zheng, Leyi Dou, Shijuan Luo, Zhenhao Ye, Chaoyuan Huang

**Affiliations:** 1 The Second Clinical School of Guangzhou University of Chinese Medicine, Guangzhou, China; 2 Department of Gastroenterology, The Second Affiliated Hospital of Guangzhou University of Chinese Medicine, Guangzhou, China

**Keywords:** *Helicobacter pylori*, bismuth quadruple therapy, prescription appropriateness, real-world, retrospective study

## Abstract

**Objective:**

Based on real-world clinical data, this study aims to evaluate the differences in eradication efficacy among various *H. pylori* treatment regimens and the impact of prescription appropriateness on treatment outcomes.

**Methods:**

This single-center retrospective study included 897 patients with confirmed *H. pylori* infection (September 2022–September 2025). Eradication rates were compared among dual, triple, and bismuth-containing quadruple therapies, stratified by prescription appropriateness per 2022 Chinese guidelines. Multivariable logistic regression and stratified analyses were performed to assess factors associated with eradication success and follow-up adherence.

**Results:**

Overall ITT and PP eradication rates were 79.04 and 90.23%, respectively. Quadruple therapy achieved the highest rates (ITT: 81.42%; PP: 93.76%), outperforming dual and triple therapies. Prescription appropriateness significantly affected eradication in the quadruple therapy group (*p* < 0.01). In quadruple therapy, female was associated with higher eradication success (OR = 2.53, 95% CI: 1.15–5.73). In the inappropriate dual therapy subgroup, amoxicillin dose (<2.0 g vs. ≥2.0 g/day) did not significantly alter eradication rates. Older patients and those using TCM demonstrated better follow-up compliance.

**Conclusion:**

Clinical practice should prioritize guideline-recommended regimens and standards; however, actual eradication effectiveness may vary depending on the regimen selected. Among these, bismuth-containing quadruple therapy should be the first-line treatment of choice and should be used in strict accordance with guideline standards.

## 1 Introduction

*Helicobacter pylori (H. pylori)* infection is highly prevalent worldwide, affecting approximately 48.9% of the global population (1). As a significant risk factor for various digestive system diseases, *H. pylori* has been classified as a Group I carcinogen by the World Health Organization (2). Eradicating *H. pylori* not only alleviates gastrointestinal symptoms but also reduces the risk of gastric cancer (3).

*H. pylori* eradication therapy is currently recommended by clinical guidelines worldwide. Commonly used regimens include bismuth-containing quadruple therapy, triple therapy, and high-dose dual therapy (4–7). These regimens have demonstrated substantial clinical efficacy and effectively reduce the risk of associated diseases (8). A study by Tan et al. published in The Lancet noted that randomized controlled trials (RCTs) are typically conducted in homogeneous populations with standardized procedures and ideal adherence. When their findings are extrapolated to real-world clinical practice, factors such as patient heterogeneity, treatment complexity, and fluctuating adherence may cause actual efficacy to deviate from expectations (9). However, increasing antimicrobial resistance in *H. pylori* has become a major concern. A survey of data from the Asia–Pacific region over the past 30 years revealed resistance rates of 52% for metronidazole, 26% for levofloxacin, and 22% for clarithromycin (10). This substantial regional heterogeneity further compounds the challenge (11), undermining the real-world effectiveness of existing regimens. Moreover, inappropriate prescribing practices have been observed in routine clinical settings (12, 13). Therefore, the comparative effectiveness of different eradication regimens and the impact of prescribing appropriateness on treatment outcomes require further validation through real-world retrospective studies.

Currently, real-world retrospective studies remain limited in several respects. First, some studies have only compared efficacy differences within individual regimens using subgroup analyses, without directly comparing multiple regimen types (14–17). Second, few studies have systematically assessed prescribing appropriateness. For instance, Shi et al. reported that insufficient drug dosage in bismuth-containing quadruple therapy significantly reduced eradication success rates (18); however, their analysis was limited to a single regimen type. In summary, real-world retrospective studies that simultaneously encompass multiple regimen types and systematically evaluate the impact of prescribing appropriateness on eradication outcomes remain scarce.

Accordingly, this study conducted a retrospective analysis using real-world clinical data to address the following key questions: first, to validate the differences in eradication efficacy among different regimens in real-world settings; and second, to compare eradication rates between appropriate and inappropriate prescriptions, thereby assessing the impact of prescribing appropriateness on treatment outcomes. Additionally, multivariable analyses were performed to account for potential confounders, including age and concomitant use of traditional Chinese medicine (TCM), along with adherence analyses, thereby strengthening the real-world evidence for optimizing individualized treatment strategies.

## 2 Materials and methods

### 2.1 Study design

This was a single-center, retrospective, observational study. Clinical data were retrieved from the electronic medical record system of the Ersha Island Branch of Guangdong Provincial Hospital of Traditional Chinese Medicine for patients diagnosed with *H. pylori* infection between September 2022 and September 2025. The study protocol was approved by the Ethics Committee of Guangdong Provincial Hospital of Traditional Chinese Medicine (Approval No. ZE2026-123-01) and conducted in accordance with the Declaration of Helsinki.

### 2.2 Inclusion and exclusion criteria

The inclusion criteria were as follows: (1) patients diagnosed with *H. pylori* infection at the outpatient department of the Ersha Island Branch of Guangdong Provincial Hospital of Traditional Chinese Medicine between September 2022 and September 2025; (2) diagnosis confirmed by a positive 13C-urea breath test or histopathological examination of gastric tissue indicating *H. pylori* positivity. The exclusion criteria comprised cases with missing key information in the medical records (e.g., medication regimens, follow-up results) and cases with duplicate records.

### 2.3 Data collection

The following data were extracted from electronic medical records: (1) demographic characteristics: age and gender; (2) clinical information: date of visit, diagnostic basis, and detailed treatment regimen (including drug names, single dose, frequency, and total duration of therapy), as well as concomitant use of TCM; (3) outcome measures: the results of the 13C-urea breath test (negative, positive, or not repeated) performed at least 4 weeks after completion of eradication therapy, along with the test date.

### 2.4 Grouping

Grouping was performed based on the 2022 Chinese Clinical Practice Guidelines for *H. pylori* Treatment (7): (1) grouping by treatment regimen: patients were categorized into the dual therapy group, the triple therapy group, and the quadruple therapy group; (2) grouping by prescription appropriateness: Within each regimen group, patients were further subdivided into an “appropriate prescription subgroup” and an “inappropriate prescription subgroup” based on whether the drug types and dosages adhered to the guideline standards. Detailed information is provided in Table 1.

**Table 1:** Drug dosages and criteria for qualified *H. pylori* eradication regimens.

Category	Components and dosages	Appropriate criteria	Inappropriate criteria
Criteria for appropriate and inappropriate regimens			
High-dose dual therapy	Amoxicillin ≥3.0 g/day + PPI at double standard dose bid (or standard dose qid)	Complete regimen: amoxicillin ≥3 g/day at correct frequency + PPI at double standard dose bid (or standard dose qid) +14-day course	Amoxicillin <3 g/day; Incorrect dosing frequency; PPI not at double standard dose bid/standard dose qid; Missing component; Non-14-day course
Triple therapy	PPI + 2 antimicrobial agents	Complete triple combination: PPI at standard dose bid + 2 antimicrobials at specified doses/frequencies +14-day course	Missing PPI or antimicrobials; PPI or antimicrobial dose/frequency non-compliant; Non-14-day course
Bismuth quadruple therapy	PPI + Colloidal bismuth subcitrate (CBS) + 2 antimicrobial agents	Complete quadruple combination: PPI at standard dose bid + CBS 220 mg bid + 2 antimicrobials at specified doses/frequencies +14-day course	Missing any component; PPI, bismuth, or antimicrobial dose/frequency non-compliant; Non-CBS bismuth; Non-14-day course

Appendix: drug components	
Antimicrobials	Amoxicillin 1 g bid
	Clarithromycin 500 mg bid
	Levofloxacin 500 mg qd or 200 mg bid
	Tetracycline 500 mg tid/qid
PPI	Metronidazole 400 mg tid/qid
	Omeprazole 20 mg bid
	Esomeprazole 20 mg bid
	Rabeprazole 10 mg bid
	Lansoprazole 30 mg bid
	Pantoprazole 40 mg bid
	Ilaprazole 5 mg bid
Bismuth	CBS 220 mg bid

### 2.5 Data processing and analysis

Complete case analysis (CCA) was used to handle missing data. During the data cleaning process, missing data were addressed according to the following rules: (1) Missing outcome variables: all patients with clearly defined treatment regimens were included in the intention-to-treat (ITT) analysis, and patients lost to follow-up or with missing outcome data were considered treatment failures. In the per-protocol (PP) analysis, only patients who completed follow-up and had available outcome data were included. (2) Missing covariates (e.g., key variables such as age, sex, and medication regimen): Given that missing data accounted for less than 5% and followed a missing completely at random (MCAR) pattern, we excluded cases with missing values instead of using imputation. Thus, bias arising from complete-case analysis is negligible. (3) Recording of reasons for non-follow-up: The specific reasons for patients not returning for re-examination were recorded in detail and categorized (including adverse reactions, busy schedule, forgetfulness, assumed eradication after medication, notification issues, and others).

Analyses were conducted in R (v4.5.1). Age was described as median [interquartile range (IQR)] and compared using the Mann–Whitney U test. Categorical variables used the χ^2^ test or Fisher’s exact test. Univariate analysis was used to screen for variables associated with *H. pylori* eradication outcomes (*p* < 0.05), which were then included in a multivariable logistic regression model. Since “prescription type-appropriateness status” encompasses “medication regimen” (dual, triple and quadruple therapy), the two variables are inherently correlated and prone to multicollinearity. We assessed multicollinearity *a priori* using the variance inflation factor (VIF), with a cutoff of VIF > 5 for severe multicollinearity. Given this inherent correlation, we developed two separate models for the respective variables. Two separate models were constructed: Model 1 included the two variables as independent predictors (separate-variable model), and Model 2 combined them into a single composite variable (combined-variable model). In the event of complete separation in either model, Firth’s correction was applied to that model. The goodness-of-fit of the two models was compared using the Akaike information criterion (AIC) and the likelihood ratio test. The model with a lower AIC and no complete separation issues was selected as the primary analysis model. Stratified regression analyses were performed by treatment regimen. In the inappropriate dual therapy subgroup, amoxicillin dose was dichotomized at 2 g/day. Results were reported as odds ratio (OR) (95% confidence interval [CI]). Two-tailed *p* < 0.05 was considered significant.

## 3 Results

### 3.1 Baseline characteristics

**Figure 1:**
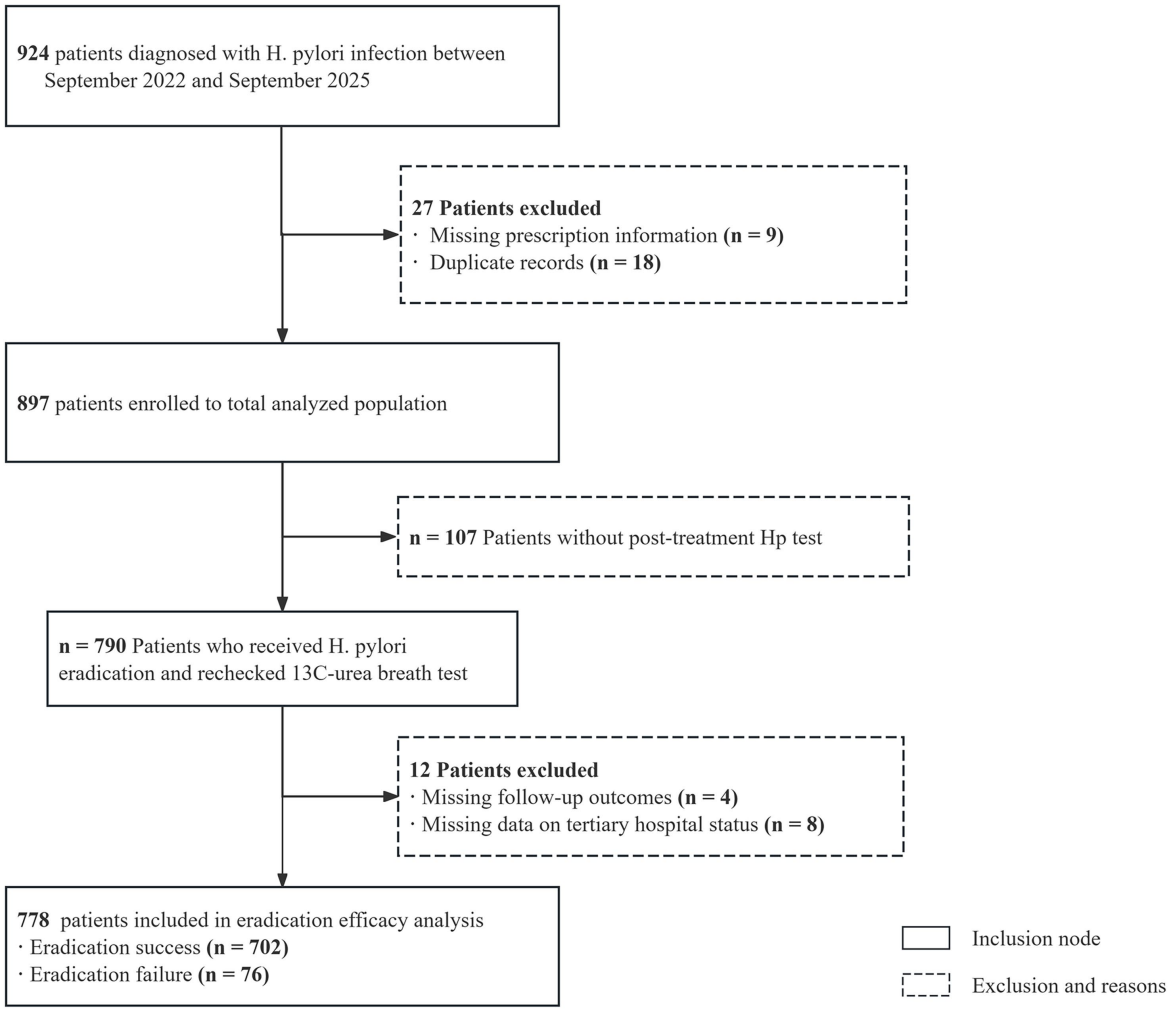
Flowchart of patient enrollment.

After screening, a total of 897 patients with *H. pylori* infection were included in the ITT analysis. The overall median age of the patients was 47 years (IQR: 37–57 years), and 336 (37.46%) were male. Among these 897 patients, 387 (43.14%) received inappropriate prescriptions, including 74 patients receiving dual therapy, 66 receiving triple therapy, and 247 receiving quadruple therapy. The specific types of prescription errors are presented in Table 2. After further excluding patients lost to follow-up and those with missing data, a total of 778 patients were finally included in the PP analysis. The detailed screening process is illustrated in Figure 1.

**Table 2:** Error type distribution by regimen.

Medication regimen	Error type	N	Percentage of regimen	Percentage of all case
All therapy	–	387	–	100
Dual therapy	–	74	–	19.12
	Antibiotic dose insufficient	64	86.49	
	PPI dose insufficient	5	6.76	
	Inappropriate antibiotic combination	3	4.05	
	Antibiotic dose insufficient + PPI dose insufficient	2	2.70	
Triple therapy	–	66	-	17.05
	Antibiotic dose insufficient	14	21.21	
	PPI dose insufficient	31	46.97	
	Antibiotic dose insufficient + PPI dose insufficient	21	31.82	
Quadruple therapy	–	247	–	63.82
	Antibiotic dose insufficient	99	40.08	
	PPI dose insufficient	1	0.40	
	Inappropriate antibiotic combination	7	2.83	
	Bismuth dose insufficient	94	38.06	
	Antibiotic dose insufficient + Bismuth dose insufficient	29	11.74	
	PPI dose insufficient + Bismuth dose insufficient	3	1.21	
	Inappropriate antibiotic combination + Bismuth dose insufficient	4	1.62	
	Antibiotic dose insufficient + PPI dose insufficient	1	0.40	
	Antibiotic dose insufficient + Inappropriate antibiotic combination	1	0.40	
	Antibiotic dose insufficient + PPI dose insufficient + Bismuth dose insufficient	8	3.24	

Significant differences were observed between the follow-up and non-follow-up groups in age and TCM usage status (*p* < 0.05). Within the follow-up cohort, age, TCM usage status, and prescription appropriateness also differed significantly among treatment subgroups (p < 0.05). To minimize confounding, these variables were adjusted for in subsequent multivariable regression analyses. Detailed baseline characteristics are presented in Table 3.

**Table 3:** Baseline characteristics of patients.

Variable	Overall (*n* = 897)	Group non-follow-up (*n* = 107)	Group follow-up (*n* = 790)					*p*-value
			Total	Dual therapy group (*n* = 268)	Triple therapy group (*n* = 66)	Quadruple therapy group (*n* = 456)	*p*-value	
Age (median [IQR], years)	47 (37–57)	39 (33–50)	48 (38–58)	48 (38–58)	41 (35.2–52.8)	48.5 (38–59)	0.0317	<0.001
Age group [*n*(%)]							0.3730	<0.001
≤30 years	117 (13.04)	20 (18.69)	97 (12.28)	35 (13.06)	10 (15.15)	52 (11.40)		
31–45 years	310 (34.56)	51 (47.66)	259 (32.78)	89 (33.21)	28 (42.42)	142 (31.14)		
46–60 years	307 (34.23)	27 (25.23)	280 (35.44)	95 (35.45)	20 (30.30)	165 (36.18)		
>60 years	163 (18.17)	9 (8.41)	154 (19.49)	49 (18.28)	8 (12.12)	97 (21.27)		
Gender [*n*(%)]							0.2769	0.5829
Male	336 (37.46)	37 (34.58)	299 (37.85)	109 (40.67)	20 (30.30)	170 (37.28)		
Female	561 (62.54)	70 (65.42)	491 (62.15)	159 (59.33)	46 (69.70)	286 (62.72)		
Traditional Chinese medicine usage status [*n*(%)]							<0.001	<0.001
Unused	669 (74.58)	97 (90.65)	572 (72.41)	153 (57.09)	53 (80.30)	366 (80.26)		
Used	228 (25.42)	10 (9.35)	218 (27.59)	115 (42.91)	13 (19.70)	90 (19.74)		
Prescription appropriateness status [*n*(%)]							<0.001	0.1270
Appropriate	510 (56.86)	53 (49.53)	457 (57.85)	210 (78.36)	10 (15.15)	237 (51.97)		
Inappropriate	387 (43.14)	54 (50.47)	333 (42.15)	58 (21.64)	56 (84.85)	219 (48.03)		
Diabetes mellitus [*n*(%)]							0.6958	0.5166
Absent	873 (97.32)	103 (96.26)	770 (97.47)	259(96.64)	66 (100.00)	445 (97.59)		
Present	24 (2.68)	4 (3.74)	20 (2.53)	9 (3.36)	0 (0.00)	11 (2.41)		
Hyperlipidemia [*n*(%)]							0.0672	1.0000
Absent	853 (95.09)	102 (95.33)	751 (95.06)	260 (97.01)	64 (96.97)	427 (93.64)		
Present	44 (4.91)	5 (4.67)	39 (4.94)	8 (2.99)	2 (3.03)	29 (6.36)		
Peptic ulcer [*n*(%)]							0.4763	1.0000
Absent	848 (94.54)	101 (94.39)	747 (94.56)	251 (93.66)	65 (98.48)	431 (94.52)		
Present	49 (5.46)	6 (5.61)	43 (5.44)	17 (6.34)	1 (1.52)	25 (5.48)		

### 3.2 Eradication efficacy of different regimens

#### 3.2.1 Comparison by treatment regimen

In the ITT analysis (*n* = 897), the overall eradication rate was 79.04%. Quadruple therapy achieved the highest eradication rate (81.42%), followed by dual therapy (76.77%) and triple therapy (71.79%). PP analysis (*n* = 778) showed a similar pattern, with quadruple therapy maintaining the highest eradication rate (93.76%), followed by dual therapy (85.23%) and triple therapy (86.15%). ITT and PP eradication rates by medication regimen are presented in Table 4.

**Table 4:** ITT and PP eradication rates by medication regimen.

Group name	ITT total cases	ITT eradication rate (%)	PP total cases	PP eradication rate (%)
All samples	897	79.04	778	90.23
Medication regimen				
Dual therapy	297	76.77	264	85.23
Triple therapy	78	71.79	65	86.15
Quadruple therapy	522	81.42	449	93.76
Prescription appropriateness status				
Appropriate	510	80.00	451	90.02
Inappropriate	387	77.78	327	90.52
Prescription type-appropriateness status				
Appropriate dual therapy	223	78.03	208	83.17
Appropriate triple therapy	12	66.67	9	88.89
Appropriate quadruple therapy	275	82.18	234	96.15
Inappropriate dual therapy	74	72.97	56	92.86
Inappropriate triple therapy	66	72.73	56	85.71
Inappropriate quadruple therapy	247	80.57	215	91.16

#### 3.2.2 Comparison by prescription appropriateness status

In the ITT analysis, the eradication rate was higher in the appropriate-prescription group than in the inappropriate-prescription group (80.00% vs. 77.78%). In the PP analysis, the appropriate-prescription group and the inappropriate-prescription group showed similar eradication rates (90.02% vs. 90.52%) (Table 4).

#### 3.2.3 Comprehensive comparison by prescription type and appropriateness status

After stratification by treatment regimen and prescription appropriateness, ITT analysis showed that the appropriate quadruple therapy group had the highest eradication rate (82.18%), followed by the inappropriate quadruple therapy group (80.57%) and the appropriate dual therapy group (78.03%). For the PP analysis, the appropriate quadruple therapy group (96.15%) remained highest; among the remaining groups, the inappropriate dual therapy group (92.86%) had the second-highest eradication rate, while the inappropriate quadruple therapy group (91.16%) ranked third (Table 4).

### 3.3 Factors associated with *H. pylori* eradication

#### 3.3.1 Univariate analysis

Among the 778 patients ultimately included in the PP analysis, univariate analysis was performed to identify factors associated with *H. pylori* eradication. The results showed that only treatment regimen (*p* < 0.001) and prescription type-appropriateness status (p < 0.001) were significantly associated with eradication outcome, whereas age, gender, TCM usage status, and comorbidities showed no significant associations. Details are provided in Table 5.

**Table 5:** Univariate analysis of factors associated with *H. pylori* eradication.

Variable	*p*-value	OR	95% CI
Age group			
≤30 years (ref)	0.8731	–	–
31 ~ 45 years		1.09	0.45–2.79
46 ~ 60 years		1.32	0.63–2.70
>60 years		1.14	0.56–2.26
Gender			
Male (ref)	0.2147	–	–
Female		1.39	0.84–2.31
Medication regimen			
Dual therapy (ref)	0.0005	–	–
Triple therapy		1.08	0.48–2.68
Quadruple therapy		2.60	1.52–4.52
Prescription appropriateness status			
Appropriate (ref)	0.9136	–	–
Inappropriate		1.06	0.64–1.77
Prescription type-appropriateness status			
Appropriate dual Therapy (ref)	0.0004	–	–
Appropriate triple Therapy		1.62	0.21–73.82
Appropriate quadruple Therapy		5.04	2.30–12.25
Inappropriate dual therapy		2.62	0.88–10.63
Inappropriate triple therapy		1.21	0.51–3.23
Inappropriate quadruple therapy		2.08	1.11–4.01
Traditional Chinese medicine usage status			
Unused (ref)	0.2355	–	–
Used		0.71	0.42–1.23
Tertiary hospital status			
Tertiary Grade A Hospital (ref)	1.0000	–	–
Non-Tertiary Grade A Hospital		1.08	0.26–9.76
Diabetes mellitus			
Absent (ref)	0.4339	–	–
Present		0.60	0.17–3.29
Hyperlipidemia			
Absent (ref)	0.7025	–	–
Present		0.72	0.27–2.44
Peptic ulcer			
Absent (ref)	0.8742	–	–
Present		0.81	0.31–2.73

(ref) indicates the reference group; CI, confidence intervals; OR, odds ratio; Statistical significance was defined as *p* < 0.05.

#### 3.3.2 Multivariable analysis

To avoid collinearity between the type of regimen (dual therapy, triple therapy, quadruple therapy) and the combined variable of regimen type and appropriateness status, two separate models were established: Model 1 (separate variable model) and Model 2 (combined variable model). The likelihood ratio test for Model 1 showed that the interaction model significantly improved model fit compared with the basic model (LRT *χ*^2^ = 15.7863, *p* = 0.0033). The Hosmer-Lemeshow goodness-of-fit test supported adequate model fit (*χ*^2^ = 3.1089, *p* = 0.9273). Assessment of multicollinearity indicated that all variance inflation factor (VIF) values were < 3.0, suggesting no severe multicollinearity. Model comparison revealed that Model 1 had a lower AIC value (489.96) than Model 2 (493.15). Therefore, Model 1 was adopted as the primary analysis model, and Model 2 was used as a sensitivity analysis to validate the robustness of the results. The main effects were largely consistent between the two models. Quadruple therapy achieved a significantly higher eradication rate than dual therapy (OR = 3.45, 95% CI: 1.49–8.49), whereas inappropriate quadruple therapy was associated with a lower eradication rate compared with appropriate dual therapy (OR = 0.15, 95% CI: 0.03–0.54).

#### 3.3.3 Subgroup analysis

**Figure 2:**
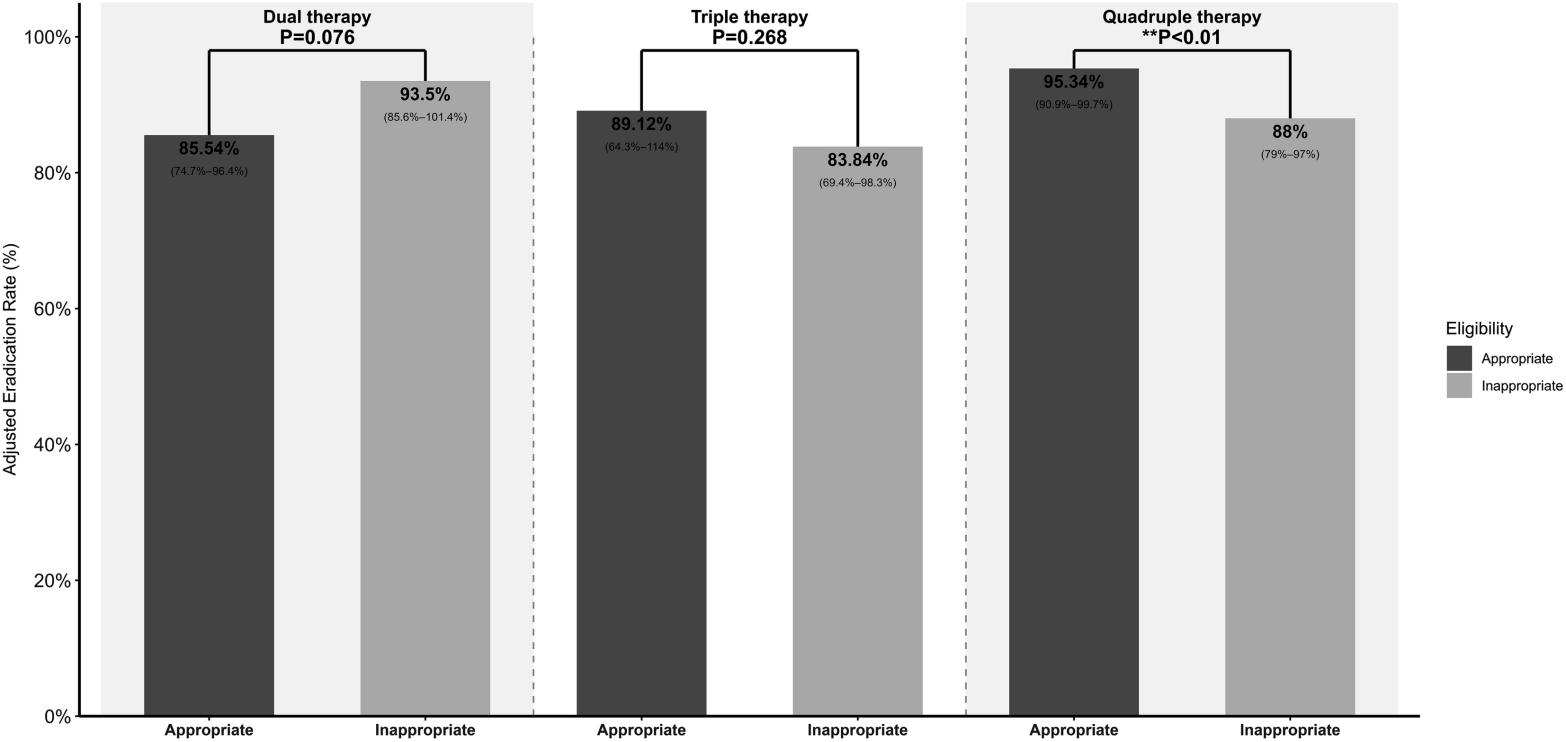
Eradication Rates of *H. pylori* by Treatment Regimen and Prescription Appropriateness. Note: Adjusted eradication rates (%) are predicted values from the model 1. Values in parentheses indicate 95% CIs. *p < 0.05, *p < 0.01, ***p < 0.001 vs. appropriate prescriptions.

Subgroup analyses were conducted within the framework of Model 1. The results showed that there was a significant difference in *H. pylori* eradication rates between appropriate- and inappropriate-prescription subgroups within quadruple therapy (*p* < 0.01). Further details are provided in Figure 2. In the quadruple therapy regimen, female patients had a notably higher eradication rate than male patients (OR = 2.53, 95% CI: 1.15–5.73). Among quadruple therapy subgroups, the eradication rate in the inappropriate-prescription group was 67% lower than that in the appropriate-prescription group (95% CI: 0.14–0.74). In the inappropriate dual therapy group (*n* = 56), 51 patients were included in the dose analysis (5 cases without amoxicillin). The eradication rate was 93.75% (95% CI: 69.77–99.84) in the group receiving a daily amoxicillin dose of <2.0 g (*n* = 16), and 91.43% (95% CI: 76.94–98.20) in the group receiving ≥2.0 g (*n* = 35). There was no statistically significant difference in the risk of eradication failure between the two groups (OR = 1.40, 95% CI: 0.10–78.65).

#### 3.3.4 Robustness of results

Wald post-hoc power analysis indicated insufficient statistical power for the comparison between the triple therapy group and the dual therapy group (5.7%), suggesting limited reliability of the estimates for the triple therapy group. To exclude the potential influence of triple therapy on the overall results, a sensitivity analysis was performed by removing the triple therapy group and re-estimating a robust model consistent with the structure of Model 1. The Hosmer-Lemeshow goodness-of-fit test indicated adequate model fit (*χ*^2^ = 14.2665, *p* = 0.0751), and the direction and statistical significance of the main effects remained consistent with those in Model 1, indicating that the core results were robust. The statistical power for the main effect of quadruple therapy was 95.6%, suggesting that the current sample size was sufficient to detect this effect. Together with its statistical significance (*p* = 0.0047), these findings support the reliability of the observed difference in eradication efficacy between quadruple therapy and dual therapy.

### 3.4 Follow-up behavior analysis

Univariate analysis showed that age and TCM usage status were significantly associated with follow-up behavior (*p* < 0.05). Detailed results are presented in Table 6. The main reasons for not returning for follow-up included being too busy (31.78%), forgetfulness (24.30%), and perceived eradication (20.56%). Multivariable analysis (Table 7) indicated that middle-aged and older patients, as well as those receiving concurrent TCM, demonstrated significantly higher adherence to follow-up testing (*p* < 0.05). Detailed results are presented in Table 7.

**Table 6:** Univariate analysis of factors associated with follow-up behavior.

Variable	*p*-value	OR	95% CI
Age group			
≤30 years (ref)	0.0004	–	–
31 ~ 45 years		3.51	1.46–9.14
46 ~ 60 years		1.05	0.56–1.90
>60 years		2.13	1.08–4.15
Gender			
Male (ref)	0.5829	–	–
Female		0.87	0.55–1.35
Prescription appropriateness status			
Appropriate (ref)	0.0923	–	–
Inappropriate		0.72	0.47–1.09
Traditional Chinese medicine usage status			
Unused (ref)	0.0001	–	–
Used		3.69	1.88–8.09

**Table 7:** Multivariate analysis of factors associated with follow-up behavior.

Variable	OR	95% CI	*p*-value
Age group			
31 ~ 45 years	0.98	0.54–1.73	0.9454
46 ~ 60 years	2.18	1.15–4.08	0.0175
>60 years	3.61	1.62–8.57	0.0015
Gender			
Female	0.94	0.60–1.44	0.7700
Traditional Chinese medicine usage status			
Traditional Chinese medicine used	3.40	1.79–7.13	0.0001

## 4 Discussion

In this study, bismuth-containing quadruple therapy showed a higher *H. pylori* eradication rate than dual therapy and triple therapy. This finding is highly consistent with the recommendations of major international and national guidelines (19). The 2022 Chinese Guideline on the Treatment of *H. pylori* Infection explicitly recommends bismuth-based quadruple therapy as the preferred choice for both initial and repeat eradication therapy (7). Similarly,the 2024 clinical guideline from the American College of Gastroenterology (ACG) emphasizes that optimized bismuth-containing quadruple therapy is the preferred regimen for treatment-naive patients when antibiotic susceptibility is unknown (4). The 2022 Maastricht VI/Florence Consensus Report also lists bismuth-containing quadruple therapy as a first-line recommendation (20). Therefore, the present study provides complementary real-world observational evidence supporting the applicability of these guideline recommendations in clinical practice.

The impact of prescription appropriateness on eradication efficacy showed marked regimen-specific characteristics: a statistically significant difference was observed only in the quadruple therapy group, whereas no significant effects were noted in the dual or triple therapy groups. This phenomenon may be explained from two perspectives: regional resistance characteristics and the different mechanisms of action of each regimen. The resistance rates in the overall Asia–Pacific region and in Guangzhou, China, share some similarities. The latest survey on antimicrobial resistance in the Asia–Pacific region reported a clarithromycin resistance rate of 30%, and a rate of 46% in Guangzhou, China (10, 21). Given this high resistance rate, major domestic and international guidelines no longer recommend clarithromycin-containing triple therapy as a first-line option, and the use of this regimen in real-world clinical practice has gradually declined. This also explains the limited sample size of triple therapy in this study, resulting in insufficient statistical power for a reliable assessment of the relationship between prescription appropriateness and eradication efficacy (22). In contrast, the resistance rate of *H. pylori* to amoxicillin remains low (<10%) in the Asia–Pacific region (10, 21). High-dose dual therapy primarily relies on the combination of amoxicillin and a proton pump inhibitor (PPI), and its bactericidal effect may be attributed to the achievement of high local concentrations of amoxicillin (23, 24). In the present study, most inappropriate dual therapy prescriptions (86%) were characterized by an insufficient daily dose of amoxicillin, despite an adequate PPI dose. Based on this finding, a possible explanation is proposed: under conditions of an adequate PPI dose, the acid-suppressive effect may help maintain the stability and antibacterial activity of amoxicillin (25), thereby partially compensating for an insufficient amoxicillin dose. Therefore, in real-world clinical practice, even when some degree of prescription deviation exists, dual therapy may still be capable of achieving eradication efficacy that is not necessarily inferior to that of appropriate prescriptions. Notably, although the eradication rate in the appropriate prescription group was numerically higher than that in the inappropriate group in this study, the difference did not reach statistical significance. This finding suggests that, within certain limits of prescription deviation, it is possible that dual therapy may maintain acceptable eradication efficacy. The advantage of quadruple therapy lies in the synergistic effect of multiple drugs (19), and this synergy is highly dependent on the appropriateness of each component in the regimen. Bismuth not only directly kills *H. pylori* but also protects the gastric mucosa and enhances the synergistic action of antibiotics (26–28). Meanwhile, PPI must be administered at adequate doses (e.g., omeprazole 40 mg twice daily) to maintain intragastric PH > 6; failure to do so leads to reduced antibiotic stability and increased *H. pylori* replication, thereby directly compromising antimicrobial efficacy (25). Any deviation—such as insufficient dosage of any drug, improper drug combinations (e.g., selecting two antibiotics with high resistance rates), or inappropriate timing of administration—may disrupt this synergistic effect. In conclusion, clinical practice should focus on ensuring the appropriateness of quadruple therapy prescriptions, including adequate doses of bismuth and PPI as well as appropriate drug combinations, in order to fully realize their synergistic advantages.

This study found that certain demographic characteristics may be associated with the efficacy of eradication therapy. In the quadruple therapy group, female patients achieved a higher eradication rate than male patients. One possible explanation is hormonal factors, whereby progesterone may mediate anti-inflammatory responses, inhibit *H. pylori* growth, and confer a protective effect (29, 30). It should be emphasized that current major international and domestic guidelines preferentially recommend bismuth-containing quadruple therapy and high-dose dual therapy as first-line regimens, whereas triple therapy is no longer the standard initial choice. Therefore, demographic characteristics such as gender and age should be considered as adjunctive reference factors in treatment decisions once a specific regimen has been selected, rather than serving as independent determinants for regimen selection.

The analysis of follow-up adherence revealed that middle-aged and older patients, as well as those receiving TCM, were more likely to demonstrate higher adherence. This may be explained by the multi-target effects of TCM, including anti-inflammatory, acid-suppressive, and gastroprotective actions, which could improve eradication rates and reduce adverse reactions (31–33). Consequently, these patients may exhibit better treatment tolerance and higher follow-up adherence.

This study has several limitations. First, it was a single-center study, which may limit the generalizability of the findings to other regions. Second, as a retrospective study, clinical information relied on electronic medical records. Some potential confounders (e.g., prior eradication history, smoking and alcohol consumption, and recent antibiotic exposure) could not be included in the multivariable analysis models due to incomplete original records. Therefore, the potential impact of these unmeasured confounders on the study results cannot be completely ruled out. Finally, the statistical power for the effect of prescription appropriateness in triple therapy was insufficient, and the current sample size was not adequate to detect their true effects stably. Larger sample studies are needed for validation.

In conclusion, clinical practice should prioritize guideline-recommended regimens and standards, but the actual eradication efficacy may vary depending on the chosen regimen. Among these, bismuth-containing quadruple therapy should be the first choice for first-line treatment and should be used strictly according to guideline standards to optimize eradication outcomes and delay the development of antimicrobial resistance.

## Data Availability

The original contributions presented in the study are included in the article/supplementary material, further inquiries can be directed to the corresponding authors.
